# BLVector: Fast BLAST-Like Algorithm for Manycore CPU With Vectorization

**DOI:** 10.3389/fgene.2021.618659

**Published:** 2021-02-02

**Authors:** Sergio Gálvez, Federico Agostini, Javier Caselli, Pilar Hernandez, Gabriel Dorado

**Affiliations:** ^1^Departamento Lenguajes y Ciencias de la Computación, Universidad de Málaga, Málaga, Spain; ^2^Instituto de Botánica del Nordeste (IBONE), Universidad Nacional del Nordeste, Corrientes, Argentina; ^3^Instituto de Agricultura Sostenible (IAS-CSIC), Consejo Superior de Investigaciones Científicas, Córdoba, Spain; ^4^Departamento Bioquímica y Biología Molecular, Campus Rabanales C6-1-E17, Campus de Excelencia Internacional Agroalimentario (ceiA3), Universidad de Córdoba, Córdoba, Spain

**Keywords:** proteins, parallel algorithm, benchmarking, pairwise alignment, database search

## Abstract

New High-Performance Computing architectures have been recently developed for commercial central processing unit (CPU). Yet, that has not improved the execution time of widely used bioinformatics applications, like BLAST+. This is due to a lack of optimization between the bases of the existing algorithms and the internals of the hardware that allows taking full advantage of the available CPU cores. To optimize the new architectures, algorithms must be revised and redesigned; usually rewritten from scratch. BLVector adapts the high-level concepts of BLAST+ to the x86 architectures with AVX-512, to harness their capabilities. A deep comprehensive study has been carried out to optimize the approach, with a significant reduction in time execution. BLVector reduces the execution time of BLAST+ when aligning up to mid-size protein sequences (∼750 amino acids). The gain in real scenario cases is 3.2-fold. When applied to longer proteins, BLVector consumes more time than BLAST+, but retrieves a much larger set of results. BLVector and BLAST+ are fine-tuned heuristics. Therefore, the relevant results returned by both are the same, although they behave differently specially when performing alignments with low scores. Hence, they can be considered complementary bioinformatics tools.

## Introduction

Currently, the Basic Local-Alignment Search Tool, BLAST (Altschul et al., 1990), is one of the most widely used algorithms to search for sequence similarities in Bioinformatics. BLAST+ has been designed to be quickly executed by standard multicore microprocessors in a hardware environment, where cores are very powerful, memory accesses are extremely fast, and there is no contention among the different cores. Unfortunately, this is not the hardware scenario for the recent manycore central processing unit (CPU) coprocessors, like the Intel Xeon Phi (Jeffers et al., 2016), TILE-Gx (Schooler, 2010) or massively parallel processor array (MPPA)-256 (De Dinechin and Graillat, 2017), where cores are less powerful, coordination between a huge amounts of threads is slow, cache sizes are small, and cache faults are heavily penalized. As an example, Intel recognizes that executing BLAST+ directly on Xeon Phi in native mode is up to 3-fold slower than using a standard Xeon E5 processor (Albert, 2015). Other implementations of BLAST specifically designed for multi- and many-core architectures, like HPC BLAST (Sawyer et al., 2019), which strictly follows the BLAST heuristic algorithm, do not improve these outcomes too much, at least when the results in Xeon Phi are compared to traditional microprocessors, like Intel Xeon E5-2670 (Brook et al., 2014).

To optimize the performance of a manycore CPU executing BLAST+ or any BLAST-like application, the internal algorithm must be adapted to use the strengths of the underlying architecture: vectorization and multithreading (Langenkämper et al., 2016). We present BLVector, a BLAST-like algorithm that works with peptide (protein) sequences. BLVector is designed specifically to use the Single-Instruction Multiple Data (SIMD) vector instructions of the Advanced Vector eXtensions (AVX)-512 instruction set, as well as its multithread capabilities. We have previously demonstrated the usefulness of optimizing bioinformatics algorithms to significantly enhance performance (Gálvez et al., 2010, 2016; Díaz et al., 2011, 2014; Esteban et al., 2013, 2018).

The main contributions of BLVector are: (i) Implementation of the Smith-Waterman (S/W) pairwise local alignment algorithm, following the Farrar’s approach with vectors of 512 bits (AVX-512); (ii) A filtering stage ensures that only promising local pairwise alignments are performed; (iii) Such filtering stage uses AVX-512 vectors for a faster execution. Applying the filter to two sequences is up to 1,000% faster than the corresponding pairwise alignment without such filtering step; (iv) Thread-level parallelism maximizes the usage of computational power available in manycore CPU systems; (v) The hits retrieved with this approach are pretty similar to those obtained with BLAST+, but the execution is faster in most situations; and (vi) Different parameters of BLVector allow dealing with a trade-off between accuracy and performance.

In this work, the design of this new heuristic is presented. A deep benchmark is carried out, based on its execution on a single Xeon Phi coprocessor and other architectures. It is important to note that, although there are newer processors, this is a proof of concept. Additionally, the Intel Xeon Phi is currently in many HPC solutions. In fact, six of the Top 25 supercomputers listed in https://www.top500.org (November, 2020) incorporate the Intel Xeon Phi technology; and this number increases when other architectures that include the AVX-512 instruction set are considered, like Intel Skylake-X, Cascade Lake and others.

## Methods

BLVector is a heuristic algorithm that follows two stages to search for peptide sequences: (i) filtering; and (ii) local pairwise alignment. Many efforts have been made to execute brute-force pairwise alignments, so BLVector focuses in the filtering stage to speed up its execution. To do this, we have created an approach that takes the most from the new SIMD AVX-512 instruction set, by applying them to both stages.

In contrast to BLAST+ (blastp), which uses a hash table to search for 3-mer substrings, BLVector uses a brute-force method to search for common 4-mer substrings between the query and the subject sequence. Using one byte per amino acid (aa) residue, BLVector packs four bytes in a simple 32-bit integer. Besides, by using SIMD vector instructions, it compares a single query integer (four aa residues from the query sequence) against 16 subject integers (16 blocks of four aa residues in the subject sequence), in parallel in a single clock cycle per instruction: 16 integers × 32 bits = 512 bits. Section “BLVector Search for 4-mer” provides further information about it.

BLVector considers that a subject sequence might be a promising hit when, at least, there are four 4-mer matches in a query window of 16 consecutive aa residues, though this can be parameterized. In such a case, BLVector executes a local alignment (Smith and Waterman, 1981) against the query, in order to determine if the subject is actually a relevant hit. To do this, a new Farrar’s implementation (Farrar, 2007) of the S/W algorithm has been developed from scratch using the AVX-512 architecture, although other approaches may also be used for such a purpose. This behavior is different to blastp, which executes a search around each 3-mer match to expand it; only when its size and score is beyond a threshold, the subject sequence is considered as a relevant hit. In addition to vectorization, BLVector uses a multiprocessing approach, taking the most from the manycore CPU architecture, by executing four threads per core (see section “BLVector Execution Architecture”).

The Farrar algorithm is an elegant method to apply vectorization to a single Smith-Waterman pairwise alignment. Based on a complex rearrangement of indices, it assumes that most cells in the dynamic programming matrix will be zero. This allow an intensive application of SIMD vector instructions, and only in a few cases is needed to execute a correction stage that penalizes the algorithm. The Farrar approach is the fastest known to perform single local alignments. It suits to the BLVector operating mode, where an alignment is performed as soon as a new promising subject sequence passes its filtering stage. This way, a single thread of BLVector interleaves the execution of the filtering and alignment stages. Other methods, like Rognes (2011), are adapted to work faster with sets of subject sequences. Regarding performance, BLVector provides three main parameters:

Nearby: Number of 4-mer matches to be found in a 16 aa-residue window of a subject sequence, to be considered as a potential hit. As this value increases, the sensitivity of BLVector decreases.

Cluster: To increase sensitivity, aa-residue letters can be grouped based on their distribution statistics^1^.

Faster: This Boolean parameter makes BLVector execute a non-exhaustive search, looking for 4-mer only in even positions.

### BLVector Search for 4-mer

The key point of the filtering implementation is the utilization of specific memory data structures. That allows to align amino acid sequence residues (letters) of peptides or proteins in memory, as required by SIMD instructions. To do this, BLVector creates four replicas of the subject sequence stored in memory; each one of them being shorter than the previous one in 1 byte (a single residue is stored into one byte; that is, 8 bits). This speeds up the comparison of a 4-mer query against every 4-mer subject, exclusively using vectors in which the computation unit is 32 bits; i.e., 4 consecutive residues or 4-mer. Although such approach requires more main memory than BLAST+, this quadruplication is executed on-the-fly, each time that BLVector is executed. Therefore, there is no need for a previous indexation stage, as is required by the former algorithm. As in many other approaches (Liu et al., 2011, 2013; Rognes, 2011; Yongchao and Schmidt, 2014). BLVector focuses on amino acid sequences because this implies the usage of relatively large score matrices, like point-accepted mutation (PAM) (Dayhoff et al., 1978) or blocks substitution matrix (BLOSUM) (Henikoff and Henikoff, 1992). Therefore, these algorithms should be used for nucleic acid (DNA or RNA) alignments, in addition to proteins, only when this type of matrices is used, as is the case of substitution matrices for aligning nucleic acid sequences using the International Union of Pure and Applied Chemistry (IUPAC) NUCleotide (NUC) ambiguity codes, like NUC.4.4.

**FIGURE 1:**
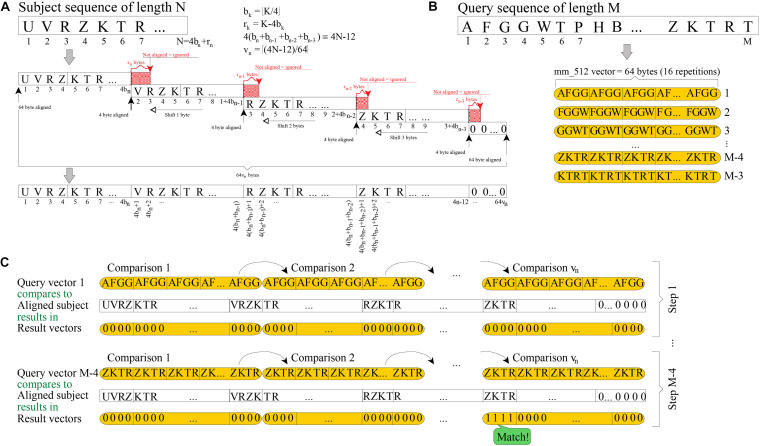
Scheme of 4-mer (32 bits) searching by using vectors of 512 bits. **(A)** Required quadruplication of a subject sequence before searching for 4-mer. **(B)** How each query 4-mer is replicated 16 times into 512-bit vectors is shown. **(C)** Each 512-bit vector is checked against each quadruplicated subject and, hence, a single query 4-mer is searched in v_*n*_ steps (16-fold faster than using non-vectorized instructions).

Figure 1A shows how a subject sequence of length N is quadruplicated into memory, using 64[(4N-12)/64] bytes. The resulting memory structure must be aligned to 64 bytes (512 bits) at both ends, because vectors of 512 bits are being used (a vector is interpreted as 16 consecutive units of 32 bits); and each replica is aligned to four bytes (the length of 4-mer; i.e., a string sequence of four residues). Quadruplication may be seen as a way to prebuild into memory all the vector values that will be needed in further processing.

The goal of the filtering stage is to check common substrings of length four, between a query sequence of length M and a previously quadruplicated subject. To do this, the BLVector algorithm focuses on each substring of four bytes (4-mer) length in the query sequence. Then, each 4-mer is replicated 16 times into a vector of 512 bit (4 bytes × 16 × 8 bits per byte = 512 bits of length; see Figure 1B. This approach allows each query’s 4-mer to be checked against the subject sequence, using only v_*n*_ = [(4N-12)/64] comparisons between vectors of 512 bits length. This is shown in Figure 1C which, in addition, shows an example of a match of the 4-mer query at position M-4. The total number of comparisons in the worst case is (M-3) v_*n*_. The vector instructions used in BLVector are compatible with those of newer x86 architectures (AVX-512F) by means of intrinsic functions^2^.

Additionally, the rationale is to check those cases where there is a local concentration of common 4-mers between the subject and the query. To track locality, BLVector uses a 16-bit integer (named nearby) that shifts to the left for each of the M-3 iterations. When one of these iterations results in a match, then the least significant bit (LSB) of nearby is set; otherwise, it is reset. Only when the number of bits set in nearby exceeds the value of the -n parameter (nearby-threshold), it is assumed that the subject has enough local similarity to the query. In such a case, it is worth to execute a local alignment between them. If the nearby variable has never contained nearby-threshold bits set after the M-3 steps, then, the subject is discarded as a potential hit.

Clearly, it can be argued that contiguous 4-mers of the query sequence may appear at very distant positions in the subject sequence. However, even in the case of random sequences, this is unlikely to happen in a section of only 16 residues, i.e., the length of the nearby integer. In the worst-case scenario, this produces a false potential hit that will be discarded after the pairwise alignment stage.

### BLVector Matrix Clustering

Usage of a 4-mer is a must in BLVector, due to the essentials of vectorization with 32-bit replicas. Other alternatives, like 2-mer (16-bit) or 8-mer (64-bit) are too loose or restrictive, respectively, for filtering purposes. Considering that blastp uses a 3-mer for residues, we have created a method for BLVector to resemble blastp: matrix clustering.

Matrix clustering consists in reducing the number of letters used in the whole algorithm; i.e., from the 21 letters used in a standard score matrix of aa residues (corresponding to the 20 natural amino acids encoded by the genetic code, plus X for unknown ones) to a minor amount. To do this, two or more letters were collapsed into a single one, following different approaches: (i) chemical families; (ii) k-means; or (iii) statistical representations. The suitability of each approach depends on the ratio between new hits and new promising hits. In other words, the average number of new pairwise alignments to be done in order to find out a new hit subject sequence. After testing these approaches, the statistical distribution of each residue available in UniProt (Bateman, 2019) was chosen as the main variable to apply a hierarchical clustering. By far, this method introduced the lesser noise and produced the best results.

The cluster parameter allows specifying the number of letters to be used in BLVector, which ranges from 21 to 15 [that, including the wildcard symbol or asterisk (^∗^), fits into a nibble]. For example, using a cluster value of 20, simply assumes that the tryptophan and cysteine amino acids refer to the same residue. The rationale is that their respective statistical distributions are 1.0 and 1.3%, being the lowest reported by UniProt. This approach has proved to be more suitable than, for example, collapsing cysteine and methionine (which belong to the same sulfur-containing family of aa) to create the cluster of 20. Using lower values of the cluster introduces a blur effect in the sequences, whose final effect is that more sequences pass the filter and need to be pairwise aligned. Hence, the accuracy is increased but at the cost of reducing the speedup. At some extent, reducing the value of this parameter has a similar effect than reducing the value of the nearby parameter, albeit at the expense of introducing some noise. In general, the best results were obtained by fine-tuning the value of nearby, and using a cluster of value 21 or 20. This can be observed in the interactive figure “BLVector_nX_cXX_X.html” (Gálvez et al., 2020) and Supplementary Material “FullMetrics.xlsx” (see section “Results and Discussion”).

### Non-exhaustive Heuristic Algorithm

Looking for the best hit is a widely used operation. Even more, this operation is usually employed to discover the main functionality of just discovered proteins, after the assembly of a new genome. In cases like this, the user searches for very similar subjects that share long substrings with the query. In other words, a very high value for the nearby parameter in valid hits is expected. Because of this, the same most relevant results may be obtained in a faster way, by comparing just the even locations of the subject sequence (instead of both, the even and odd). Only the most promising sequences will pass the filter. So, the number of pairwise alignments to perform will decrease (including invalid hits), and the execution time of BLVector will be dramatically reduced.

For this purpose, we propose to shorten the replication of the subject sequence: a duplication is used instead of a quadruplication. This is achieved by using the fast parameter -f. Thus, only the original sequence and its copy shifted 2 bytes are used in the filtering stage. Therefore, the time taken by 4-mers searches is divided by two. The drawback of this approach is that searches for 4-mer are not exhaustive, because they are looked for at even positions only. Clearly, this is a risky heuristic algorithm that should be used only when the user is looking for very similar proteins, and where the probability of skipping common 4-mer is not a problem to find the required hits. Using this method, the average gain in giga cell updates per second (GCUPS) is ∼58.7%, whereas the loss of hits is ∼49.7% (see Supplementary Material “FullMetrics.xlsx”).

### BLVector Execution Architecture

A typical execution of BLVector begins loading the fast-alignment sequence tools (FAST)-All (FASTA) (Lipman and Pearson, 1985) database into memory, further replicating the sequences, as stated in Figure 1A. In this stage, the random-access memory (RAM) memory may be a bottleneck; loading a RAM disk file into the RAM application memory takes 2 s per GiB. Hence, retrieving the Swiss-Prot UniProt file (∼250 MiB) into memory takes ∼0.5 s. Once the file has been loaded into memory, several threads are in charge of quadruplicating the sequences. However, from 10 threads on, no additional speed gain is obtained, due to contention to main memory accesses. Thus, the final result is that this operation is twice slower than file loading: takes ∼1 s to quadruplicate the Swiss-Prot UniProt. When only a few query sequences will be searched against the database, it is much more efficient to quadruplicate queries, instead of the whole database of subjects. Nonetheless, in this work we provide the particular (and more general) approach. Thus, it is assumed that users will search for many thousands of sequences and, thus, taking the most of the performance of BLVector in big-batch jobs.

**FIGURE 2:**
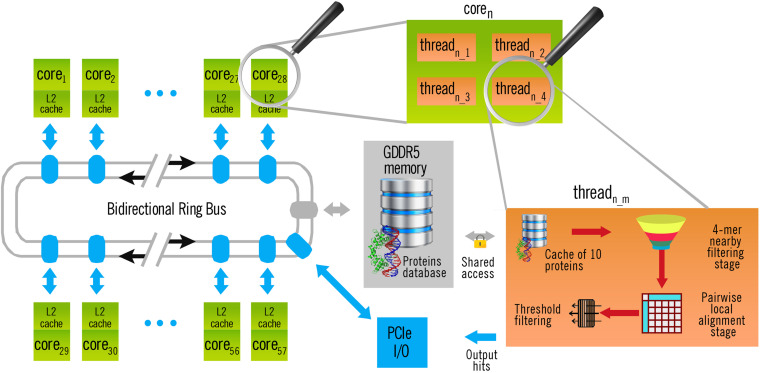
Architecture of BLVector execution in a Xeon Phi Coprocessor 31S1P with 57 cores. Each thread executes a 4-mer nearby filtering stage, as indicated in Figure 1.

The left part of Figure 2 shows the general architecture of the Xeon Phi Coprocessor 31S1P used in this work. As the memory bus is shared among all the cores, memory-intensive tasks produced a bottleneck when they could not be fully executed inside the cache. Fortunately, this whole stage is executed only once, independently of the number q of query sequences to search for in a single execution. Yet, the time taken by this operation tends to be insignificant as q increases. Thus, it makes no sense to include it in the calculus of performance (see section “Results and Discussion”).

Once each subject sequence is quadruplicated into a memory pool, the query file is also loaded and split into single FASTA sequences. Then, for each query sequence, and one after the other, the BLVector heuristic algorithm starts 228 threads (this can be changed through the -p parameter), like the one illustrated at the right of Figure 2. This is a requirement of the Xeon Phi Coprocessor 31S1P, that contains 57 cores and requires to use 4 threads per core to obtain the maximum performance. When a thread becomes idle, it blocks the pool where the subject sequences are stored, it takes the next ten ones to process and unblocks the pool. All the threads work with the same query sequence, but against different subjects stored in the main memory: filtering them and aligning only those ones that passes the filter (see right-bottom part of Figure 2). Taking ten sequences at each step (instead of only one) reduces threads contention and, at the same time, maintains a balanced workload amongst them. BLVector leverages this approach to noticeably increase performance. Actually, the optimal size of this local cache of proteins largely depends on the length of the query sequence (contention increases with shorter sequences), and the final value of 10 is empirical. In any case, each thread processes a subject sequence at a time (whose average length is 359 residues in Swiss-Prot UniProt release 2019_05). Therefore, the memory footprint is small, and memory-intensive operations are executed inside its own cache.

The success of BLVector is mainly due to the high performance obtained through the AVX-512 SIMD instructions used in the 4-mer nearby filtering stage, although vectorization has also been used in the pairwise alignment stage. In addition, unrolling four times the main loop of this 4-mer search (by means of a #pragma), also slightly increases the performance. In short, looking for 4-mer using AVX-512 instructions is much faster (see section “Results and Discussion”) than using a hash table in standard microprocessors, as BLAST+ does. Finally, a correct alignment of data into memory is extremely important to achieve a good performance in the execution of the Smith-Waterman algorithm with affine gaps (Gotoh, 1982), using the stripped approach of Farrar (Farrar, 2007). Other approaches could be used to align many subject sequences with extreme performance. However, they do not include a filtering stage and/or they require specific servers and/or high-end graphics cards, whose cost greatly exceeds the affordable Xeon Phi Coprocessor 31S1P that BLVector may use (Lan et al., 2017; Rucci et al., 2019). Anyway, the BLVector source code could be rewritten to get the most out of any brand new hardware vectorization.

## Results and Discussion

The main contributions of this implementation are the utilization of a filtering stage to reduce the amount of pairwise alignments to perform, and the efficient use of the 512-bit vectors provided by the AVX-512 instruction set, both in the filtering and alignment stages. Avoiding contention to shared memory accesses also contributes to increased efficiency.

The BLVector has been tested in native mode, on a Xeon Phi Coprocessor 31S1P with 8 GiB DDR5 and 57 cores at 1.1 GHz, running 228 threads, with a thermal design power (TDP) of 270 W. It is quite affordable, with a current cost of around 200 USD. This card supports a 512-bit instruction set, named initial many core instructions (IMCI); i.e., a precursor of some AVX-512 extensions (including AVX-512F), being compatible with them by means of intrinsic functions. The behavior study of BLVector is divided into three parts: (i) performance and accuracy against blastp, including a real case of study; (ii) improvement introduced by the filtering stage; and (iii) speedup of executing the filtering stage in different architectures.

### Performance and Accuracy of BLVector

BLVector has been used to search for the 32 proteins reported elsewhere (Rognes, 2011) using the Swiss-Prot UniProt release 2019_05 (560,292 sequences with an average length of 359 residues). Using default parameters, execution times ranged from <1 s for sequences shorter than 730 aa residues to 9.2 s for proteins like P33450 (5,147 aa residues). BLVector can be considered as a Smith-Waterman alignment algorithm, with a former filtering stage that passes promising subject sequences only. Hence, we have chosen the concept of GCUPS to measure its performance against blastp (BLAST+ v2.7.1), that has been executed in two different personal computers (PC): (i) a desktop PC with an Intel Core i7-4820K CPU running at 3.70 GHz, 12 GiB of DDR3 and a Kingston HyperX 3K Solid-State Drive (SSD; 120 GB); and (ii) a workstation with an Intel Xeon W-2123 (Skylake microarchitecture) CPU with 4 cores running at 3.60 GHz, 32 GiB of DDR4 and a Corsair Force MP510 NVMe SSD (500 GB). For comparison purposes, BLVector has been executed inside the Xeon Phi card with no support of the main CPU, whereas blastp has been fully executed in the main CPU, with no support of the Xeon Phi card. The performance of the Intel i7-4820K and Xeon W-2123 used in this experiment are very close to that of the Intel Xeon E5-2670 used in other studies (Orozco-Arias et al., 2017; Sawyer et al., 2019). Therefore, the scenarios where BLVector and blastp have been executed are completely standard, and thus comparison fairness is guaranteed. In this sense, each program is executed in its native homogeneous hardware architecture. The performance of blastp is slightly better in the workstation, so it is used in the benchmarks of this discussion (see the Supplementary Material “FullMetrics.xlsx” for more information).

**FIGURE 3:**
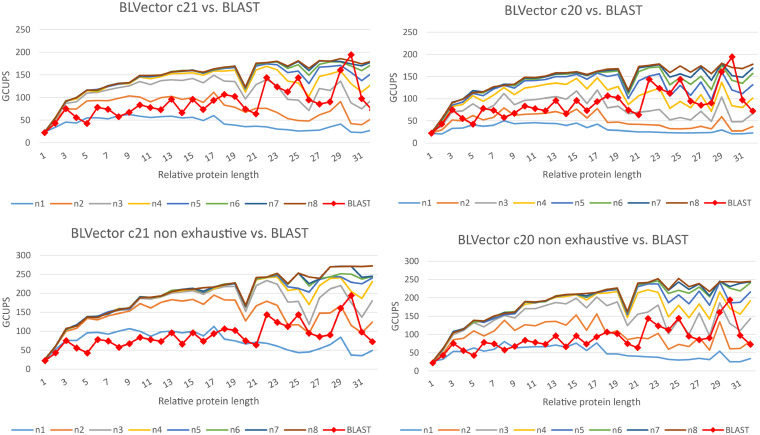
Speed performance of BLVector with 228 threads vs. BLAST+ using BLOSUM62. BLAST+ times are the same in every chart. The top charts show executions of BLVector with parameter -n from 1 to 8 and -c 21 (left) and -c 20 (right). The bottom ones represent the same executions, but including the non-exhaustive parameter -f. Execution speeds are represented in GCUPS. As an example, in the top-left diagram, BLVector uses cluster c21 and nearby values from n1 to n8; the speed of BLVector exceeds BLAST+ from n3 on.

BLVector and BLAST+ are heuristics, and they may return different sets of hits, in comparison to an optimal Smith-Waterman whole search. Hence, their performances should be compared for both hits retrieved (accuracy and precision), as well as execution times (speed). Different parameter values were used to benchmark BLVector time performance in comparison to blastp (see Figure 3 and Supplementary Material, for benchmarks with different matrices from the BLOcks SUbstitution Matrix 62 – BLOSUM62). The best ones were selected as default ones.

**FIGURE 4:**
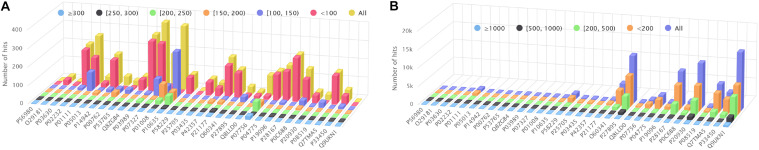
Hits performance of BLVector vs. BLAST+ using BLOSUM62. *X* axis shows the names of the proteins used for benchmarking. **(A)** How many hits given by BLAST+ are not given by BLVector, using -n 3 and -c 21 parameters. These hits are divided into six sets, depending on the bitscore given by BLAST+, ranging from <100 to ≥300. **(B)** BLAST+ (blastp) misses thousands of hits correctly returned by BLVector, with proteins larger than 1 kb (see vertical axis labels). These hits are divided into four sets, depending on the score given by BLVector.

Figure 4A shows the amount of hits found by BLAST+ (blastp), but not by BLVector (see the interactive figure “BLVector_Nx_Cxx_X.html” (Gálvez et al., 2020) for hits retrieved by BLVector in contrast to BLAST+, when using different sets of parameters). In general, BLVector misses tens of hits whose bit-score is lower than 200; i.e., those less relevant, and this happens mainly for the shortest proteins. In contrast, when dealing with long proteins, larger than one kilobase (kb), BLAST+ misses thousands of hits, which are correctly returned by BLVector; see Figure 4B and interactive figure “BLAST_all_hits_vs_BLVector.html” (Gálvez et al., 2020).

**FIGURE 5:**
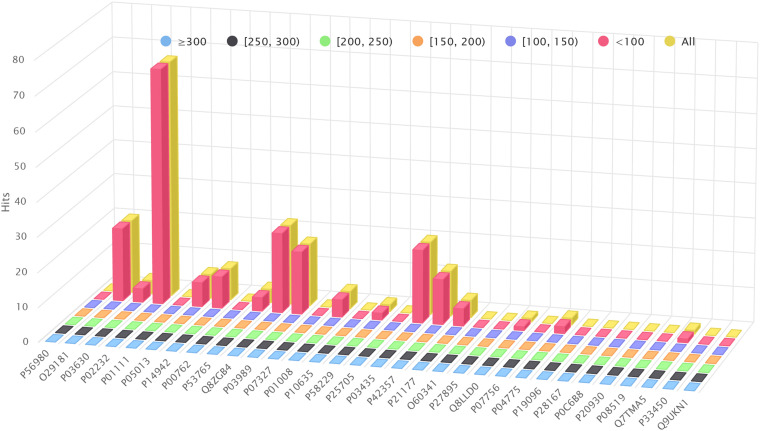
Number of hits retrieved by BLAST+, but not by a Smith-Waterman execution. BLOSUM62 and default parameters have been used in both cases; Smith-Waterman was set with a threshold score of 80.

Figure 5 shows how many hits reported by BLAST+ are skipped by a pure Smith-Waterman algorithm (i.e., removing the nearby filtering stage of BLVector; see the interactive figure “Farrar.html” (Gálvez et al., 2020) for more details). It reveals that BLAST+ provides hits even below the default threshold used by BLVector (score of 80 using BLOSUM62). The interactive figure “BLVector_nX_cXX_X.html” (Gálvez et al., 2020) must be studied taking these results into account. Thus, it displays the hits skipped by BLVector with different parameters: -n from 1 to 8, -c from 15 to 21 and activating/deactivating -f. The corresponding and most interesting execution times, expressed in GCUPS, are illustrated in Figure 3, and the complete set can be found in the Supplementary Material “FullMetrics.xlsx.” All times and GCUPS values are the average results of three independent executions, under the same conditions, including default parameters. BLVector may achieve higher performance with short proteins, by reducing the number of threads to avoid contention. In other words, BLVector is heavily penalized when using a large number of threads, like the default value of 228, for short sequences. For large sequences, this default value obtains the best results and, in addition, uses the resources of the coprocessor at full load.

In a real world scenario, an all vs. all search was executed between the primary proteins of the cereal genomics model grass *Brachypodium distachyon* 314 reference genome (v3.1 containing 34,310 protein sequences and 12,944,043 aa residues, with an average length of ∼377 aa residues per protein^3^; and the ∼32,000 primary proteins of the drought-stress tolerant variety ABR8 (containing 32,609 protein sequences, with an average length of 366 residues) (Fisher et al., 2016). These proteins are relatively short, so the time taken by BLVector to start 228 threads was even more that the time used to process a single protein. Hence, a best performance was achieved with a lower number of threads per protein. Using five simultaneous executions of BLVector, each one with 44 threads (parameters as -p 44 -n 3 -c 21 -f), consumed just 628 s to execute the whole task, instead of 2,022 s taken by blastp (parameters as -num_threads 8 -outfmt 6 -max_target_seqs 1 -max_hsps 1). That represents a performance gain of 3.22-fold with the same relevant output results. The performance achieved by each BLVector instance execution was relatively uniform, ranging between 49.2 and 49.5 GCUPS with a wattage consumption of ∼160 W (∼92 W when idle), a constant user CPU usage of ∼93% and a memory utilization of 1.32 GiB.

### The Filtering Stage and Its Speedup

It is important to estimate how much time saves BLVector, when compared to a whole S/W execution. When a sequence is rejected in the filtering stage, no local pairwise alignment should be executed. So, the total time to process such a sequence has been the filtering time. On the other hand, when the filtering stage foregoes a sequence, the local alignment must be executed, so the total time to manage the sequence is the filtering plus the alignment times.

**FIGURE 6:**
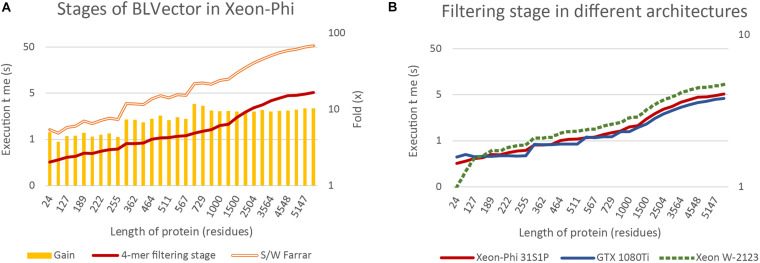
BLVector execution times in relation to sequence length. Both vertical axes are logarithmic and *X* axis shows the lengths of the proteins used for benchmarking. **(A)** Benchmarks of the 4-mer nearby filtering stage versus the corresponding Smith-Waterman execution in the Xeon-Phi. **(B)** A comparison of the filtering stage execution times among different architectures: Xeon-Phi (31S1P), CUDA (GTX 1080Ti) and Skylake (Xeon W-2123).

Figure 6A benchmarks the times of two theoretically extreme executions of BLVector: i) pure Smith-Waterman (Farrar, 2007), without filtering stage; and (ii) pure 4-mer nearby filtering stage, without Smith-Waterman execution (nearby of ∞). The filtering stage using 512-bit vectors is approximately one order of magnitude faster than the execution of the Farrar algorithm. The gain achieved by applying the filtering stage (represented by bars) can be split in three main blocks: (i) up to the average length of proteins in Swiss-Prot UniProt 2019_05 (359 aa) with a gain of ∼5×; (ii) from 361 aa up to 567 aa with a gain of ∼7×; and (iii) from 657 aa on with a gain of ∼10× (a maximum of 11.8× is achieved on P42357, with a length of 657 aa). This implies that the overload of applying the filtering stage to a hit protein is around 10% whereas the time saved when applied to a non-hit protein is between 500 and 1,000%. Logically, there is a direct relationship between the speedup gain and the percentage of filtered sequences that, in turn, depends on the parameters of BLVector (see the Supplementary Material “FullMetrics.xlsx” and “Charts SW”).

### The Filtering Stage in Other Architectures

By far, the most relevant part of BLVector is the filtering stage and its performance. For this reason, it is important to compare its throughput when executed in other widespread hardware architectures. Figure 6B includes a comparison of the filtering stage (nearby of ∞, i.e., maximum accuracy and execution time of this stage) executions times among different architectures, including CUDA (Ryoo et al., 2008). The graphics card used for this test has been a GTX 1080Ti with 11 GiB of GDDR5X running 3584 cores at 1.48 GHz and a TDP of 250 W. It must be noted that the implementation in CUDA of the BLVector filtering stage does not require the quadruplication of the data because the vectorization used in Xeon-Phi is translated into simpler memory accesses. The proteins used in this benchmark are allocated into CUDA shared memory, and this significantly improves the speed up. In addition, the memory unalignment problem is resolved in CUDA by using 32-bit integers shifted 8 bits in each new comparison, achieving, this way, a minimum number of memory accesses in the inner loop of the algorithm. With the outstanding resources of the card used (62.8× more cores, 1.38× more memory and 1.35× faster), the CUDA approach achieves only a gain of ∼25% in the best cases.

The BLVector filtering stage behaves extremely fast when executed on manycore CPU systems with small cache sizes, many threads per core and heavily penalized accesses to main memory. For comparison purposes, it is advisable to test how the filtering stage behaves when executed on a workstation CPU. Thus, the BLVector filtering stage has been recompiled in an Intel Xeon W-2123 (Skylake microarchitecture) CPU with 4 cores running at 3.60 GHz and 32 GiB of DDR4 RAM memory, as well as a Corsair Force MP510 NVMe SSD (500 GB). This is one of the simplest AVX-512 capable Intel processors, with only 4 cores and a TDP of 120 W. Figure 6B shows also the behavior of the filtering stage in this CPU. This implementation scales exactly the same than in Xeon-Phi, though it is ∼1.6× slower with a 44.4% of its TDP. Finally, a complete execution in the real case scenario explained in section 0 consumed just 909 s (average of three executions), only a 45% slower than the Xeon Phi and 2.22-fold faster than blastp executed in the same CPU. This result shows that the AVX-512 set of instructions has a really promising future when correctly used, and its usage in current supercomputers with these SIMD instructions should enhance its performance.

## Conclusion

As said, the BLVector is a heuristic algorithm like BLAST+ (blastp), searching for local similarities as a filtering step prior to perform S/W executions. Because it relies on local alignments, BLVector is halfway between a pure heuristic and a pure brute-force method. This work shows that modern algorithms must be designed focusing on current CPU architectures to obtain optimum performances, instead of older, traditional architectures. That can be accomplished by using the simplest portion of the recent x86 vector instructions set: AVX-512F. In addition, the design is simplified using two stages, a front-end filter and a back-end pairwise aligner, both based on vectorization. Deep benchmarking analyses reveal that BLVector is a fast algorithm that provides accurate results. And, in real world scenarios, it produces the same results than blastp, but faster. Filtering a sequence through the 4-mer method is up to 11.8-fold quicker than calculating its S/W score, so it can be applied not only to Farrar algorithm, but also to any brute-force approach (Rognes, 2011; Rucci et al., 2019), as a way to speed it up.

In a future work, several coprocessors may be used to benchmark BLVector on larger databases; e.g., the Viridiplantae portion of the Translation of the European Molecular Biology Laboratory (TrEMBL) amino acid sequence database (∼5 GB), splitting it into parts and compounding results as in Gálvez et al. (2016). To avoid many splits of the database, quadruplication of query subjects may be an option in order to save memory. In addition, a new version of S/W –using AVX-512BW– providing also sequence alignments would be useful. BLVector can be easily used in blastx mode; i.e., translating a nucleotide sequence from double-stranded DNA (dsDNA) or double-stranded RNA (dsRNA) into their six representations of amino acids (reading frames), to address scenarios where only nucleic-acid sequences are available. Finally, it will be very interesting to know the behavior of BLVector when implemented through the recent Intel oneAPI in order to use not only CPU but also GPU with minor efforts. To do this, the alignment stage implementation based on Farrar algorithm can be substituted by that of Rognes (2011).

## Data Availability Statement

Publicly available datasets were analyzed in this study. This data can be found here: https://www.uniprot.org (Swiss-Prot UniProt 2019_05) and https://phytozome.jgi.doe.gov (*Brachypodium distachyon* 314 reference genome). The source code of BLVector is available at https://github.com/galvezuma/BLVector.

## Author Contributions

All authors participated in the development, performed testing and analyses, as well as the writing of the manuscript, and revised the final version.

## Conflict of Interest

The authors declare that the research was conducted in the absence of any commercial or financial relationships that could be construed as a potential conflict of interest.

## Footnotes

1 https://www.uniprot.org/statistics/Swiss-Prot 2019_05

2 https://software.intel.com/sites/landingpage/IntrinsicsGuide

3 https://phytozome.jgi.doe.gov
